# Intussusception in a 4-Year-Old Male Due to Burkitt Lymphoma

**DOI:** 10.1155/2023/3535164

**Published:** 2023-03-27

**Authors:** Raed Al-Taher, Abdallah Alabadla, Salameh Al-Halaseh, Ghasaq Saleh

**Affiliations:** ^1^Department of General Surgery, Division of Pediatric Surgery, School of Medicine, University of Jordan, Amman, Jordan; ^2^School of Medicine, University of Jordan, Amman, Jordan

## Abstract

Intussusception is the invagination of a proximal bowel segment into a distal segment causing bowel obstruction, especially in children. In some cases, it can be caused by a pathological lead point, such as Burkitt lymphoma. Burkitt lymphoma has several patterns of clinical presentations, such as jaw or facial bone tumor in the endemic form, in contrast to an abdominal presentation most often with massive disease and ascites. We describe a case of a 4-year-old male who presented bowel obstruction. Using X-ray and ultrasound findings, ileocecal intussusception was then diagnosed. Resection and anastomosis was performed after multiple trials of failed hydrostatic reduction. On the pathology report of the resected segment, Burkitt lymphoma was found to be the cause and chemotherapy was initiated. The patient is doing well and is following up every 6 months for 2 years. A pathological lead point, especially Burkitt lymphoma, should be suspected in patients with failed conservative treatment, and prompt diagnosis of the pathology should be performed to prevent further sequela of the disease.

## 1. Introduction

Intussusception is an acquired invagination (telescoping) of a proximal bowel (intussusceptum) into a distal bowel segment (intussuscipiens); it is a relatively common cause of bowel obstruction in children. In a national-wide study in Switzerland, the mean yearly incidence of intussusception was 38, 31, and 26 cases per 100,000 live births in the first, second, and third years of life, respectively. The incidence decreases after the third year of life to less than one-half of these rates [1].

Intussusception is classified according to its location, entero-enteric, entero-colic, and colo-colic. Ileocolic intussusception accounts for 80% of the cases [2].

The etiology of pediatric intussusception is usually idiopathic, with only 10% of cases having an identifiable precipitating lesion [3]. Intussusception can be caused by a variety of congenital conditions, including Meckel's diverticulum, intestinal duplication, lesions including polyps and hamartomas, or cancers (lymphoma and carcinoma from juvenile polyposis syndromes), and many other etiologies were described in the literature [3].

Non-Hodgkin lymphomas are the most common extranodal lymphomas, and the ileum has the highest gut-associated lymphoid tissue which makes it the most common place for lymphoma to occur in [4]. Due to the high proliferation rate of Burkitt lymphoma, it is characterized histologically by a “starry sky” appearance indicating a high rate of mitosis and apoptosis of these tumor cells [5].

Treatment of limited stage (stage I and II) Burkitt lymphomas is usually very successful, with a long-term survival rate of over 90% [6].

## 2. Case Description

We present a case of a previously healthy 4-year-old male who presented to our hospital with recurrent bouts of colicky abdominal pain, which progressed in severity over two weeks. Additionally, the patient suffered from persistent vomiting causing a significant weight loss of 6 kg (from 24 kg to 18 kg in two months). On physical examination, the patient was conscious, alert, and well-oriented. The abdomen was soft with no tenderness; the patient was initially diagnosed by the pediatric department with amoebiasis after a positive stool antigen test and was treated with oral 15 mg/kg metronidazole for 10 days and antimotility agents; however, his symptoms failed to resolve. The stool antigen test was also positive for *Helicobacter pylori* infection and he was started on triple therapy of daily oral omeprazole, amoxicillin, and clarithromycin, with no improvement in his symptoms.

Nine days later, the child was admitted again to our hospital as a case of intractable vomiting for adequate rehydration and control of the vomiting. An abdominal X-ray was performed and the impression of fecal impaction was made which contributes to his symptoms as the findings of the X-ray were insignificant; subsequently, he was discharged on laxatives.

Two days later, the patient presented again with persistent vomiting; a chest and abdomen X-ray and an ultrasound were performed subsequently as part of the management workup.

**Figure 1:**
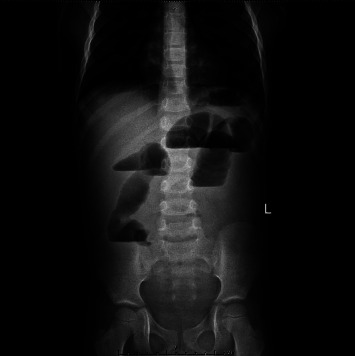
Abdominal X-ray. X-ray showing multiple air-fluid levels, which is suggestive of bowel obstruction.

**Figure 2:**
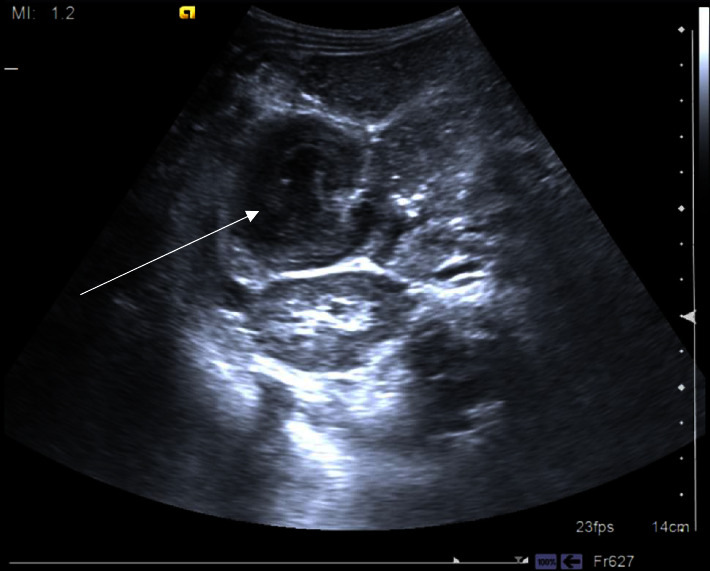
Abdominal ultrasound. Ultrasound showing target sign (arrow), which is suggestive of intussusception.

Abdominal X-rays of the upper abdomen (Figure 1) revealed dilated small bowel loops at the left upper abdomen that measured 4 cm in diameter. Moreover, ultrasound images (Figure 2) showed signs of complete intestinal obstruction, target/pseudo-kidney sign which can be seen at the right midabdomen and is suggestive of ileocecal intussusception, in association with dilated fluid-filled proximal small bowel loops.

After conservative treatment with ultrasound-guided hydrostatic reduction failed three times, diagnostic laparoscopy was performed and showed the ileocecal segment reaching the hepatic flexure. After laparoscopic reduction failed, the procedure was converted to a laparotomy through a small vertical midline incision at the umbilicus, and manual reduction was performed in hope to alleviate the intussusception. However, more difficulty was faced during the reduction in the ileocecal region. This led us to the careful examination of the cecum which revealed mural thickening and resistance to the flow of intestinal content. We performed an ileocecal resection with hand-sewn end-to-end ileo-ascending colon anastomosis. Correction of a left inguinal hernia by ligation of the left patent processes vaginalis was also performed.

Postoperatively, the patient had an uneventful recovery and was admitted to the pediatric intensive care unit for close observation until postoperative day 2 where he was afebrile and tolerated a regular diet. The patient was then transferred to another tertiary hospital for the completion of the lymphoma treatment.

The histopathology of the resected ileocecal segment was consistent with Burkitt lymphoma. The tumor cells were positive for CD20, CD10, C-MYC, and BCL-6 (weakly positive). They were negative for CD5, CD3, BCL-2, TDT, and MUM-1. Bone marrow biopsy revealed normal cellular bone marrow with trilineage hematopoiesis and no morphological evidence of involvement by lymphoma.

Our patient was classified as stage II Burkitt lymphoma and was referred for evaluation and initiation of chemotherapeutic treatment. He was treated with two cycles of CHOP (cyclophosphamide, vincristine, prednisone, and doxorubicin) and tolerated them well.

Since the completion of the chemotherapy, the patient visited the clinic for follow-up appointments every 6 months for 2 years, according to the oncologist's protocol and he was doing well.

## 3. Discussion

Intussusception is the second most common cause of acute abdomen in children second to appendicitis [7]. Approximately, 60% of children with intussusception are under the age of one year, and 80% to 90% are under the age of two [7]. It is usually primary (no specific lead point identified) in infants aged 9–24 months, whereas children older than 3 years old are more likely to have an underlying specific lead point; common lead points can include Meckel's diverticulum, polyps, IgA vasculitis, and recent infection with adenovirus [8].

The incidence of non-Hodgkin's lymphoma acting as a lead point in intussusception is reported to be as high as 17% and even higher (more than 50%) in children over 4–6 years of age [9–11].

The classic presentation of intussusception in infants is a triad of intermittent cramping abdominal pain, bloody stools (“currant jelly” dark red stools), and a palpable sausage-shaped mass in the right abdomen [3]. This classic presentation accounts for only 15% of cases [12]; patients may also draw their legs to the chest to ease the pain [3].

Our patient did not present with these exact symptoms, but insidiously with recurrent bouts of vomiting and abdominal pain, which may be due to recurrent milder bouts of partial obstruction. This caused a delay in the diagnosis; the patient presented 4 times with the same complaint until the diagnosis of intussusception was made.

The significant weight loss experienced by patients also should worry the physicians about an underlying malignancy, which was evident in our patient.

Imaging studies to diagnose intussusception include ultrasonography which can identify features specific to intussusception such as “target” or “doughnut” sign in the transverse view and “pseudo-kidney” sign in the longitudinal view; these findings were present in our case which led us to the diagnosis [7].

However, the presence of extensive air in the distended bowel loops limits its accuracy [13]. Computerized tomography is considered the most sensitive radiology method to confirm the diagnosis of intussusception with an accuracy of 58–100% [14, 15].

Burkitt lymphoma accounts for 34% of non-Hodgkin lymphomas that occur in childhood and has a male predominance [16]. The endemic form affects children of African descent and is mostly localized in the mandibular and maxillary bones [5]. Non-African children more frequently present with the abdominal form of Burkitt lymphoma. This form tends to occur in the bowel [5].

Predisposing risk factors for lymphomas include autoimmune diseases such as celiac disease (gluten-sensitive enteropathy), infections such as EBV, HHV-8, HIV, and HTLV-1, family history, and radiation exposure [17]. All these risk factors were absent in our medically free patient.

Undiagnosed Burkitt lymphoma may present with nonspecific symptoms such as nausea, abdominal pain, vomitting, palpable mass, intestinal obstruction due to bowel compression, acute appendicitis or in our case, intussusception. This makes the diagnosis of Burkitt lymphoma more difficult [16].

Although diagnosis may be difficult, the treatment has promising outcomes as children with totally resected early stage (I or II) Burkitt lymphoma have a four-year event-free survival (EFS) rate of 98% and a four-year overall survival rate (OS) of 99% with multiagent chemotherapy agents [18]. In this case, we have presented that our patient had colicky abdominal pain, persistent vomiting, and weight loss without bloody stools or a palpable abdominal mass.

## 4. Conclusion

The gold standard treatment for intussusception due to underlying Burkitt lymphoma is laparotomy resection and anastomosis. Therefore, Burkitt lymphoma should be considered as a pathological lead point when faced with a child who presents with intussusception that fails to improve with conservative management.

## Data Availability

Data sharing is not applicable to this article as no datasets were generated or analyzed during this study.
